# Health Behavior Changes During COVID-19 Pandemic and Subsequent “Stay-at-Home” Orders

**DOI:** 10.3390/ijerph17176268

**Published:** 2020-08-28

**Authors:** Gregory Knell, Michael C. Robertson, Erin E. Dooley, Katie Burford, Karla S. Mendez

**Affiliations:** 1 Department of Epidemiology, Human Genetics, and Environmental Sciences, The University of Texas Health Science Center at Houston (UTHealth), Houston, TX 77030, USA; karla.mendez@uth.tmc.edu; 2 Center for Pediatric Population Health, The University of Texas Health Science Center at Houston (UTHealth), Dallas, TX 75390, USA; 3 Children’s Health Andrews Institute for Orthopaedics and Sports Medicine, Plano, TX 75024, USA; 4 Department of Health Promotion and Behavioral Sciences, The University of Texas Health Science Center at Houston (UTHealth), Houston, TX 77030, USA; michael.c.robertson@uth.tmc.edu (M.C.R.); erin.dooley@uth.tmc.edu (E.E.D.); kathryn.burford@uth.tmc.edu (K.B.); 5 Department of Behavioral Science, University of Texas MD Anderson Cancer Center, Houston, TX 77030, USA; 6 Michael & Susan Dell Center for Healthy Living, The University of Texas Health Science Center at Houston (UTHealth), Austin, TX 78701, USA

**Keywords:** COVID-19, physical activity, sleep, health behaviors, alcohol use, marijuana use, tobacco use

## Abstract

The COVID-19 pandemic, and resultant “Stay-at-Home” orders, may have impacted adults’ positive health behaviors (sleep, physical activity) and negative health behaviors (alcohol consumption, drug use, and tobacco use). The purpose of this study was to investigate how these health behaviors changed (increased/improved or decreased/worsened) at the early stages of the pandemic, what participant characteristics were associated with health behavior changes, and why these behavioral changes may have occurred. A convenience sample of 1809 adults residing in the United States completed a 15-min self-report questionnaire in April and May 2020. Multinomial logistic regressions and descriptive statistics were used to evaluate how, for whom, and why these health behaviors changed. Participants were primarily female (67.4%), aged 35–49 years (39.8%), college graduates (83.3%), non-tobacco users (74.7%), and had previously used marijuana (48.6%). Overall, participants primarily reported a decrease in physical activity, while sleep and all of the negative health behaviors remained the same. Changes in negative health behaviors were related (*p* < 0.05) to sex, age, parental status, educational status, job status, BMI, and depression scores. Changes in positive health behaviors were related (*p* < 0.05) to sex, parental status, job status, and depression scores. Having more time available during the pandemic was the most commonly cited reason for changing health behaviors (negative and positive). Public health efforts should address the potential for long-term health consequences due to behavior change during COVID-19.

## 1. Introduction

On 11 March 2020, the World Health Organization reached consensus that COVID-19, the respiratory disease cause by the SARS-CoV-2 virus, had reached pandemic proportions [1]. Subsequently, the United States (U.S.) issued a national state of emergency, and in March and April state governments enacted sweeping “Stay-at-Home” orders to curb the spread of the disease. The specifics of these initiatives varied by state, but they generally included advisories to stay home, bans on large gatherings, restricted access to parks and community resources, closure of schools and non-essential businesses, and quarantine orders [2]. The consequences of the COVID-19 pandemic and initiatives required to address it have been associated with marked social and environmental changes writ large, but it is not clear how these circumstances have impacted health-related behaviors that reflect public health priorities.

Unhealthy lifestyle behaviors are responsible for an enormous share of premature death and burden of disease in the U.S. [3]. Population-based longitudinal studies have emphasized the critical importance of behavioral determinants of health, including physical activity, tobacco use, alcohol consumption, substance abuse, and sleep patterns [4,5,6]. Authoritative sources have put forth public health guidelines pertaining to these behaviors [7,8,9,10,11], but adherence to recommended guidelines is not optimal. According to self-report measures, over 1/3 of U.S. adults do not meet recommended physical activity levels [12], 1/3 regularly do not meet sleep guidelines [13], 1/4 report binge drinking [14], 1/5 report using tobacco products [15], and 1/6 report using cannabis or illegal drugs [16]. Increasing adherence to recommended guidelines for these behaviors is a public health priority [17], but one of the many challenges to achieving guidelines is that behavioral lapses can lead to longer-term departures from adherence [18,19,20].

Early evidence suggests that the COVID-19 pandemic may be exacerbating sub-optimal adherence to recommended health behavior guidelines. Physical activity levels may be decreasing [21], perhaps in part due to disruptions in daily routines and the widespread closure and/or restricted access to avenues of leisure-time physical activity (such as fitness facilities and community resources). Widespread work-from-home initiatives may also be negatively impacting occupation- and active transport- related physical activity, and experts warn that COVID-19 may lead to large scale increases in sedentary behavior [22]. Early evidence suggests that the COVID-19 pandemic is negatively impacting sleep patterns, possibly owing to increased anxiety and depressive symptoms [23,24]. Early evidence also points to moderate-to-severe stress and economic insecurity experienced at a large scale [25,26,27], and experts warn of the potential for crises related increases in the consumption of alcohol, tobacco products, and other potentially addictive substances [28,29,30].

More data is needed to better characterize how the COVID-19 pandemic may be affecting health-related behaviors, and, importantly, to provide insight regarding perceptions of why, specifically, these changes may be taking place. A clearer picture may help public health professionals better remedy the pernicious downstream effects of the COVID-19 outbreak, as well as contribute to our broader understanding of processes of health behavior change in times of adversity. The aims of this study were (1) to describe how positive health behaviors (physical activity, sleep) and negative health behaviors (alcohol use, tobacco use, drug use) changed (increased/improved or decreased/worsened or stayed the same) during a 6- to 8-week period from early March to mid-April 2020; (2) to understand who were more likely to change their health behaviors, and (3) to describe reported reasons why participants were changing their health behaviors.

## 2. Materials and Methods

### 2.1. Study Design, Setting, and Participants

Data for this analysis came from a convenience sample of adults residing in the U.S. Potential participants were recruited through a digital flyer that asked interested individuals to complete an anonymous online survey on their health and related behaviors during the COVID-19 pandemic. Recruitment was completed through the investigators’ social media platforms (e.g., Facebook, Twitter, Instagram) from 15 April to 5 May 2020. Approximately 47,796 social media users viewed or interacted with the social media postings during the recruitment period, of which a total of 2766 (5.8%) agreed to participate and met the eligibility criteria (aged over 18 years, and currently residing in the U.S.). After consenting to participate (*n* = 2440), a total of 240 (1.0%), participants did not provide a sufficient level of data to be included in further analysis (i.e., did not include data on state of residence, ethnicity, race, sex) and therefore were dropped from the analysis. Finally, to remove the potential effect of COVID-19 illness on the behavioral changes under study, those that reported a confirmed or suspected COVID-19 illness (*n* = 391, 1.1%) were not included in the analysis. This resulted in an analytic sample of *n* = 1809. 

### 2.2. Data Collection

The survey took approximately 15 min to complete, and participants were free to skip any questions they did not wish to answer and could stop participating at any time. Data were collected and managed using Research Electronic Data Capture (REDCap), electronic data capture tools hosted at The University of Texas Health Science Center at Houston [31,32]. REDCap is a secure, web-based software platform designed to support data capture for research studies, providing (1) an intuitive interface for validated data capture; (2) audit trails for tracking data manipulation and export procedures; (3) automated export procedures for seamless data downloads to common statistical packages; and (4) procedures for data integration and interoperability with external sources.

### 2.3. Measures 

The self-reported survey queried the following behavioral domains: physical activity, sleep, and substance use (marijuana use, alcohol consumption, tobacco use). Specifically, physical activity was measured using the International Physical Activity Questionnaire-short form (IPAQ-short), and the time-frame queried was the past seven days [33]. Participants reported the number of days and time spent per day walking and in moderate- and vigorous- intensity activity. Per the IPAQ scoring guidelines, the sum of weekly minutes engaged in physical activity were multiplied by the respective Metabolic Equivalent Task (MET) value (walking: 3.3 METs; moderate intensity: 4.0 METs; vigorous intensity: 8.0 METs). This value was used to categorize participants into one of three levels of physical activity: low, moderate, and high. A high level of physical activity was defined as performing a combination of walking, moderate or vigorous intensity activities for 7 or more days and accumulating at least 3000 MET minutes a week. A moderate level of physical activity was defined as performing a combination of walking, moderate or vigorous intensity activities for 5 or more days completing and accumulating at least 600 MET minutes a week. Those below the threshold for moderate were classified as having low levels of physical activity [34].

Sleep quality was measured using the Pittsburgh Sleep Quality Index (PSQI) [35]. The participant was asked to report on sleep metrics *since the coronavirus outbreak* began. The PSQI consists of 19 self-rated questions, grouped into seven component scores, to assess sleep quality, such as estimates of sleep duration, latency, frequency, and severity of specific sleep-related issues. Per the PSQI scoring guidelines, each component was weighted equally on a 0–3 scale, and the seven component scores were summed to obtain a global PSQI score (range = 0–21), with higher scores indicating worse sleep quality. Additionally, participants rated their overall sleep quality as very good, fairly good, fairly bad, or very bad.

Substance use was measured using items adapted from the Behavioral Risk Factor Surveillance System (BRFSS). Briefly, participants first reported their lifetime use of each of the substances (marijuana, alcohol, and tobacco). If the participant indicated he/she had ever used the substance, a follow-up question asked if he/she had used the substance within the past 30-days, and if so, on how many days they consumed/used the substance. For the alcohol-related questions, those that reported currently consuming alcohol were further asked to report his/her average daily number of drinks.

To investigate if the COVID-19 pandemic was related to changes in these behaviors (physical activity, sleep, substance use), the survey included follow-up questions asking if their engagement in that behavior had increased/improved, decreased/worsened, or stayed the same since the pandemic began. Depending on the responses to this question, a second follow-up question asked the participant why his/her behavior changed. The *check all that apply* response options varied slightly depending on the context of the question but were generally related to responsibilities, resources (time and finances), worry/anxiety/stress, loneliness/happiness, and health concerns. 

Finally, the survey included items pertaining to sociodemographics (age (18–34 years; 35–49 years; 50 years and older), sex, race/ethnicity (non-Hispanic white, non-white), number of children in the home (zero; one or more), educational status (not a college graduate; college graduate or more), employment status (employed or student, unemployed or other)), comorbidities (zero; one or more), body mass index categories (BMI (calculated from reported height in feet and inches and weight in pounds and categorized as normal, overweight or obese)), and other health-related measures (symptoms of depression (none or mild; moderate or severe using the PHQ-9), disability status (zero; one or more)) [36]. Questions pertaining to the COVID-19 pandemic and subsequent “Stay-at-Home” policies included how many weeks the participant had been living under a “Stay-at-Home” policy, and for how many hours he/she spent at home each day on average.

### 2.4. Statistical Analysis

We conducted random forest-based imputation of missing data [37,38]. This is a nonparametric approach that assumes data are missing at random and is comparable to other commonly used approaches to handling missing data [39]. Descriptive statistics (frequencies with proportions, means with standard deviations (±SD), and medians with interquartile range (IQR)) were conducted on participant characteristics, participant behaviors, and reported reasons for changing behaviors and reported appropriately. How (increased/improved, decreased/worsened) the negative health behaviors (tobacco use, marijuana use, and alcohol consumption) and positive health behaviors (sleep quality, and physical activity) changed (Aim 1) were reported overall and by participant characteristics and tested for differences using chi-square tests, Fishers exact tests, t-tests, and Kruskal–Wallis tests with the nominal *p*-value set at 0.05. Multinomial logistic regressions were used to determine what participant characteristics were associated with changing negative and positive health behaviors (Aim 2). The relative odds of changing the negative and positive health behaviors were evaluated with the “no change” group as the referent category. The fully adjusted models were mutually adjusted for the other factors of interest (sex, age, race/ethnicity, number of children in the home, education status, employment status, disability status, BMI, comorbidity status, “Stay-at-Home” duration (weeks), average time spent at home daily (hours), and depression status). Participants’ reasons cited for changing their health behaviors during the pandemic (Aim 3) were evaluated and reported with descriptive statistics.

Missing data imputation was performed in R 3.6.2 (R Core Team, Vienna, Austria 2019). All statistical analyses were performed using Stata 16.0 (StataCorp. 2019. Stata Statistical Software: Release 16. StataCorp LLC: College Station, TX, USA). This study was reviewed and approved by the University of Texas Health Science Center’s Committee for the Protection of Human Subjects (IRB number: HSC-SPH-20-0346).

## 3. Results

A total of 1809 participants were included in the analytic sample. The majority of participants (65.6%) resided in Texas, and the remaining 34.4% resided in one of 49 other states, with no other state making up greater than 5% of the sample. Of those, 67.4% were female, 39.8% were aged 35–49 years, 83.3% were college graduates, and 81.3% were employed or students. On average, participants reported their community being under a “Stay-at-Home” order for 3.9 (±0.9) weeks, and during that time spent a median (IQR) 23.0 (21.0–23.0) hours per day at home. Nearly 20% of the sample (18.5%) were classified as experiencing moderate-to-severe symptoms of depression. See Table 1 for more detail on participant characteristics.

Descriptive statistics of the positive and negative health behaviors this sample of US adults engaged during the early stages of the pandemic are shown in Supplement Table S1. Among the negative health behaviors (tobacco use, marijuana use, and alcohol consumption), the majority of the sample report to have never used tobacco (74.7%), while 48.6% having formerly engaged in marijuana use, 12.7% reported current marijuana use, and 21.7% reported consuming one alcoholic drink per day on average. Among the positive health behaviors (physical activity and sleep), the median (IQR) of MET minutes per week was 2034.0 (891.0–4464.0). More than two-thirds of the participants were classified as achieving either high (45.4%) or moderate (30.6%) levels of physical activity. The global PSQI scores ranged from 1 to 19, with an overall sample mean (±SD) of 6.5 (±3.3) (a PSQI score of >5 is considered the evaluative standard for poor sleep quality) [40]. Regarding the self-evaluations of sleep quality, nearly three-quarters of the participants reported having at least fairly good sleep quality; 14.1% reported “very good” sleep quality, and 58.0% reported “fairly good” sleep quality.

How positive and negative health behaviors changed (increased/improved, decreased/worsened, or stayed the same) during the observation period is reported in supplement Table S2 (negative health behavior) and S3 (positive health behavior) and summarized in Table 2. Overall, there were significant differences in the proportions of reported changes in health behaviors. Among the negative health behaviors (tobacco use, marijuana use, and alcohol consumption), participants primarily reported these behaviors stayed the same (50.3%, 53.0%, and 49.6%, respectively). Among the positive health behaviors, a greater percentage of participants reported decreased physical activity (39.0%) than increased (25.2%) or remained the same (35.8%). Meanwhile, the majority of participants reported their sleep quality remained the same (59.4%). 

Table 3 presents who were more or less likely to change their negative health behaviors during the pandemic. This was estimated by calculating the relative odds of changing negative health behaviors by participant characteristics (age, race/ethnicity, number of children in the home, educational status, disability status, BMI, comorbidities, time under “Stay-at-Home” order, time spent at home, and depression score). All estimates were mutually adjusted for the other participant characteristics. Changes in tobacco use were related to educational status, job status, sex, and age. Specifically, those with a college education (OR = 0.29 (95% confidence interval (CI) = 0.10–0.80)), and those classified as unemployed or other job statuses (OR = 0.11 (95% CI = 0.02–0.58)), had significantly lower odds of reporting a decrease in their tobacco use compared to their respective counterparts (not college graduates, employed or student job status) after controlling for other factors of interest. Female participants had 2.5 times greater relative odds (OR = 2.46 (95% CI = 1.10–5.47)) of reporting an increase in tobacco use compared to males after controlling for other factors of interest. Those aged 50 years and older had significantly lower odds (OR = 0.31 (95% CI = 0.10–0.92)) of increasing tobacco use compared to those aged 18–34 years after adjusting for other factors of interest. Changes in marijuana use were associated with symptoms of depression. Those with moderate- to severe- symptoms of depression had significantly higher odds (OR = 3.15 (95% CI = 1.58–6.25)) of increasing marijuana use compared to those with no symptoms of depression after adjusting for other factors of interest. Changes in alcohol consumption were related to age, educational status, BMI, number of children, and depression scores. Specifically, those aged 35–49 years (OR = 0.49 (95% CI = 0.30–0.78)) and 50 years and older (OR = 0.46 (95% CI = 0.28–0.77)), college graduates (OR = 0.46 (95% CI = 0.30–0.71)), those who are overweight/obese (OR = 0.62 (95% CI = 0.43–0.90)) had significantly lower odds of decreased alcohol consumption compared to their counterparts, after controlling for other factors of interest. Alternatively, those in the oldest age group (age 50 years or more) had 0.46 times the relative odds (OR = 0.54 (95% CI = 0.38–0.78)) of increasing alcohol consumption compared to those aged 18-34 years, after controlling for other relevant factors. While those with children (OR = 1.58 (95% CI = 1.19–2.09)) and those with a moderate to severe depression symptom severity score (OR = 2.24 (95% CI = 2.41–4.64)) had significantly higher odds of an increase in alcohol consumption compared to those with none to mild depression symptom severity score.

Table 4 presents who were more or less likely to change their positive health behaviors during the pandemic. This was estimated by calculating the relative odds of changing positive health behaviors by participant characteristics, with mutual adjustment for the other relevant factors. Changes in sleep quality were related to sex, depression symptom severity scores, and job status. Specifically, compared to males, females had 1.42 (95% CI = 1.11–1.83) times the odds of reporting a worsened sleep quality after controlling for other relevant factors. Those with a moderate-to-severe depression symptom severity score had 5.32 (95% CI = 4.01–7.06) times the relative odds of reporting a worsened sleep quality after controlling for other relevant factors. Compared to those employed or students, those unemployed or with other job statuses had 0.53 (95% CI = 0.31–0.90) times the relative odds of improved sleep quality after controlling for other relevant factors. Changes in physical activity were related to sex, number of children, and depression score. Specifically, compared to males, females had 1.47 (95% CI = 1.12–1.93) times the relative odds of increasing physical activity after controlling for relevant factors. Those with children had 1.42 (95% CI = 1.07–1.90) times the relative odds of increasing activity compared to those without children. Finally, those with a moderate to severe depression symptom severity score had 5.32 (95% CI = 3.73–7.58) times the relative odds of reporting a decrease in physical activity compared with those with a none or mild depression score, after controlling for other relevant factors. Alternatively, those with a moderate to severe depression symptom severity score had 2.42 (95% CI = 1.61–3.64) times the relative odds of reporting an increase in physical activity compared with those with a none or mild depression score, after controlling for other relevant factors.

The reported reasons and motivations for changing negative and positive health behaviors are presented in supplements Tables S3 and S4 and summarized in Table 5. Participants gave a total of 6065 reasons why they believed their behavior had changed during the COVID-19 pandemic. Of those, there were only 316 (5.2%) reasons given for decreasing negative health behaviors, and 1597 (26.3%) reasons given for increasing negative health behaviors. More specifically, “health concerns” was the most commonly cited reason for decreasing negative health behaviors overall (107/316 = 33.9%) and specifically for decreasing tobacco use (24/63 = 38.1%), marijuana use (8/29 = 27.6%), and alcohol consumption (75/224 = 33.5%) (see Table S3). Meanwhile, “boredom” (375/1597 = 23.5) and “available time” (431/1597 = 27.0%) were the most commonly cited reasons given for increasing negative health behaviors overall and specifically for increasing tobacco use (64/119 = 53.8%), marijuana use (108/246 = 43.9%), and alcohol consumption (634/1232 = 51.5%) (see Table S3).

Of the reasons for changing positive health behaviors (*n* = 4152), a larger proportion of participants reported increasing positive health behaviors (2216/4152 = 53.4%) than decreasing positive health behaviors (1936/4152 = 46.6%). “More time available” was the most commonly given reason for increasing positive health behaviors overall (930/2216 = 42.0%), and specifically for increasing physical activity (359/1499 = 23.9%) and sleep duration (421/717 = 58.7%). Among the reasons given for decreasing physical activity and sleep duration, “motivation” (434/1361 = 31.8%) and “worry/stress” (370/575 = 64.3%) were the most commonly cited reasons, respectively (see Table S4).

## 4. Discussion

This study describes how positive and negative health behaviors changed during the COVID-19 pandemic and subsequent “Stay-at-Home” orders time period (March–April 2020), who was more likely to change their behaviors, and for what reasons these changes may have occurred, among a convenience sample of U.S. adults. Statewide movement restriction orders have led to immediate social and economic challenges for individuals [2], all of which have potentially adverse effects to health behaviors. Some groups appear more vulnerable to these disruptions, as this study found that among this sample of U.S. adults, females, those with children, and those with moderate-to-severe depression symptom severity were more likely to report increased engagement in negative health behaviors (alcohol consumption and tobacco use) potentially due to increases in available time or additional stressors due to competing responsibilities between family and work. Further, females and those with moderate to severe depression symptom severity reported worsened sleep quality, which may be due to increased feelings of worry and stress. Conversely, females also reported an increase in physical activity, which was most commonly attributed to increased available free time and/or boredom. These results provide preliminary understanding for the behavioral health consequences of COVID-19 beyond the clear immediate toll to physical health and human life. 

Of the reasons provided for changing health behaviors during the pandemic, over one third (36.5%) of the reported reasons for behavior change were related to increased engagement in positive health behaviors (physical activity, sleep), which was most commonly attributed to more time available and stress relief (61%). As this sample consists of relatively higher-educated, employed adults, respondents may have been able to engage in alternative forms of physical activity, such as online fitness classes or purchasing their own exercise equipment [41]. In the current study, females were more likely to increase physical activity, which could be related to stress relief/management as about 18% of reasons for increasing positive health behaviors were related to stress relief. As has been shown elsewhere, certain physical activities (e.g., yoga) have been shown to be an effective activity for changes in stress-related psychological outcomes [42]. Among females living with a male, this may also be related to a more equal division of household labor that allows females more time for leisure activities like physical activity [43]. Whereas in 2019, American women spent more time doing housework and less time engaging in physical activity than American men on any given day [44]. Those with children also reported increased activity. Parents may be finding alternative ways to engage children with physical activity in the outdoors to maintain social distancing, such as family walks or hikes, due to closures of schools and organized sports [45]. In addition, less time spent commuting to the workplace may have led to more available time to pursue physical activities. For example, approximately 40 min per day could be added as workers may no longer be commuting to work [46]. 

Interestingly, in contrast, almost as many of the overall reasons for changing behavior were related to decreasing engagement in positive health behaviors (31.9%). Feelings of worry/stress (30.9%), less motivation (22.4%), and resource concerns (23.5%) were highly reported as the reasons for this adverse behavior change. Those with moderate to severe depression symptom severity were most likely to report decreasing their physical activity behavior. Previous existing evidence also shows significant reductions in U.S. adult’s physical activity during this time period, particularly among those who are Hispanic, lower income, and those not working [21]. Although not statistically significant, non-white and unemployed participants had higher odds of reporting decreased activity in this sample. These similar findings may indicate changes in depression symptom severity may impact changes in physical activity to a greater degree. Thus, efforts are needed not only to increase opportunities for physical activity, as transport- and occupation-related physical activity are likely thwarted due to the pandemic but to also promote mental health resources, such as mindfulness training [47] and access to telehealth care providers [48].

Similarly, out of the 1597 reasons participants reported for increasing negative health behaviors, the most common reasons were reported as boredom and more time available (50.5%). Boredom has been shown to be associated with substance abuse and depression symptoms [47]. While this study did not explore these associations, those living with moderate-to-severe depression symptom severity scores had over 2.2 times higher odds of increased alcohol consumption and three times higher odds of increased marijuana use than those with none to mild depression symptom scores. Further, those with moderate-to-severe depression symptom severity scores revealed increases in other adverse behaviors such as reporting over five-times higher odds of decreasing their physical activity and worsened sleep quality than those with none to mild depression symptoms scores. Similarly, a study conducted in Australian adults during COVID-19 found higher depression symptom severity was associated with worse health behaviors—less physical activity and sleep, more smoking and alcohol drinking [49]. The current findings expand upon this recent study by additionally including specific reasons for behavior change. Elevated population-level depression prevalence due to the pandemic is a public health concern. In representative U.S. samples prior to COVID-19, about 7-8% of U.S. adults had been found to have moderate-to-severe depressive symptoms using the PHQ-9 [18,19]; however, 18.5% of this study sample was categorized as having moderate-to-severe symptoms during the pandemic using the same measure. This high prevalence of moderate-to-severe depression symptom severity is similar to those found in Australia (19.1%) [49] and China (16.8%) [25]. 

Increases in engagement with negative health behaviors and worsened sleep quality were also seen across a range of sociodemographic factors. College graduates had 1.5 higher odds of increasing alcohol consumption. Alcohol abuse during the pandemic is a concern [28]; as evidenced by 13% of the current sample drinking four or more alcoholic beverages per day. Females were two and half times more likely to increase their tobacco use and 1.4 times more likely to report feeling like their sleep quality had worsened, compared to males in this sample. Those with children were 1.6 times more likely to report increasing alcohol consumption. With the closures of schools, child cares, and summer programs, many parents have had to take on child care needs in addition to completing work responsibilities from home [50]. Nearly 44% of the sample had children, leading many to have to balance work and home responsibilities, or even quit their jobs to take on caregiving duties [51]. In addition, middle-age and older-adults were less likely than younger adults to change their tobacco use, marijuana use, and alcohol consumption. These results may be partially explained by younger adults being the most likely to be furloughed or lose work and income during COVID-19 [52,53]. Similarly, unemployed individuals in this sample were less likely to report decreasing their tobacco use and less likely to report improved sleep. These behaviors may be a result of the high U.S. unemployment rate (14.7% by April 2020), and the almost 50% of Americans reporting a loss of income [54].

While this study shows a number of reasons for changes to health behaviors, increases in stress and anxiety due to COVID-19 have been of increased concern in the recent literature [25,49] as anxiety and stress are risk factors for substance abuse behaviors and poor sleep quality [55,56,57]. Specifically, females, younger adults, lower income, and lower education have been found to have higher stress scores during COVID-19 [49], which may explain our findings. We found approximately 20% of the reported reasons for changing health behaviors were related to feelings of being more worried/stressed. The current study adds to Stanton et al. (2020) by suggesting behavior change coping mechanisms may further differ across specific subpopulations and should be further explored in future research [49]. As COVID-19 continues to spread and full or partial lockdowns remain, access and promotion of mental health services and interventions are critical to preventing increases in negative health behaviors or decreases in positive health behaviors.

The current study’s limitations should be noted. Generalizability is limited due to a convenience sample that is primarily represented by women, non-Hispanic white, and highly educated individuals. The use of an online-only survey that utilized social media platforms for recruitment may have limited participation. Therefore, these findings should not be extrapolated to any other groups of interest and future research should aim to overcome this limitation by including larger, more representative samples. However, the convenience sampling strategy provided the opportunity to provide initial, hypothesis generating information on an important and rapidly evolving topic. In addition, the self-reporting of behaviors may have led to social desirability bias; however, participants were assured of their anonymity prior to agreeing to participate in this survey. Finally, these data are cross-sectional. Although changes to behavior were compared to life prior to the coronavirus outbreak, recall bias may be an issue as local “Stay-at-Home” order duration was 3.8±1.0 weeks at time of survey completion; however, some participants (n = 143) were unsure of their time under a “Stay-at-Home” order, which was significantly (*p* < 0.05) associated with age, employment status, disability status, comorbidities, and depression scores. These were treated as missing and imputed as described. Future research should examine these health behaviors in a more representative U.S. sample in addition to measuring changes over time with follow-up surveys.

## 5. Conclusions

Aside from the COVID-19 pandemic’s severe proximal physical health consequences, long-term health consequences due to changes in health behaviors is an important public health concern. This study found that among a convenience sample of U.S. adults, particularly females and those with moderate to severe depression symptom severity were more likely to report increased engagement in adverse health behaviors (tobacco use and alcohol consumption) and worsened sleep during COVID-19, which may be due to increased available time, stress, and worry. As there is potential for health crises related to these behaviors, this study suggests public health efforts should promote health-enhancing behaviors in lieu of health-compromising behaviors during the COVID-19 pandemic.

## Figures and Tables

**Table 1:** Participant characteristics among a sample of adults residing in the US during the COVID-19 pandemic, 2020.

Characteristic	N(%)
**Total**	1809 (100.0)
**Sex**	
Male	589 (32.6)
Female	1220 (67.4)
**Age**	
18–34	570 (31.5)
35–49	720 (39.8)
50+	519 (28.7)
**Race**/**ethnicity**	
Non-Hispanic white	1483 (82.0)
Non-white ^a^	326 (18.0)
**No**. **of children**	
0	1022 (56.5)
1 or more	787 (43.50)
**Educational status**	
Not a college graduate	302 (16.7)
College graduate or more	1507 (83.3)
**Job status**	
Employed or student	1470 (81.3)
Unemployed or other ^b^	339 (18.7)
**Disability status**	
0	1669 (92.3)
1 or more	140 (7.7)
**Body mass index**	
Normal	738 (40.8)
Overweight or obese	1071 (59.2)
**Comorbid condition[s]**	
None	1100 (60.8)
1 or more	709 (39.2)
**Local stay-at-home order duration**	
Weeks, mean (SD)	3.9 (0.9)
**Time spent at home**	
Hours, median (IQR)	23.0 (21.0–23.0)
**Depression score ^c^**	
None or mild	1474 (81.5)
Moderate or severe	335 (18.5)

Abbreviations: IQR, interquartile range; SD, standard deviation. Note: ^a^ Includes American Indian/Alaska Native, Asian, Native Hawaiian or other Pacific Islander, Black or African American, more than one race, and unknown/not reported. ^b^ Includes homemaker/stay-at-home parent, retired, and unable to work. ^c^ Estimated using the Patient Health Questionnaire-9 (PHQ-9), and categorized based on 0–5: none/mild; >5: moderate or severe.

**Table 2:** How positive and negative health behaviors changed among a sample of US adults during the COVID-19 pandemic and subsequent “Stay-at-Home” orders and policies, 2020.

Negative and Positive Health Behaviors	*n* (%)	*p*
**Tobacco use**		<0.01
Increased	54 (30.5)	
Decreased	34 (19.2)	
Stayed the same	89 (50.3)	
**Marijuana use**		<0.01
Increased	84 (36.5)	
Decreased	24 (10.4)	
Stayed the same	122 (53.0)	
**Alcohol consumption**		<0.01
Increased	521 (38.5)	
Decreased	161 (11.9)	
Stayed the same	672 (49.6)	
**Physical activity**		<0.01
Increased	455 (25.2)	
Decreased	706 (39.0)	
Stayed the same	648 (35.8)	
**Sleep quality**		<0.01
Improved	175 (9.7)	
Worsened	560 (31.0)	
Stayed the same	1074 (59.4)	

**Table 3:** Relative odds of changing negative health behaviors among a sample of US adults during the COVID-19 pandemic and subsequent “Stay-at-Home” orders, 2020.

	Relative ^a^ Odds of Changing Health Behavior, OR (95% CI).					
	Tobacco Use		Marijuana Use		Alcohol Consumption	
	Decrease	Increase	Decrease	Increase	Decrease	Increase
**Sex**						
Male	ref.	ref.	ref.	ref.	ref.	ref.
Female	0.37 (0.13–1.06)	2.46 (1.10–5.47)	0.46 (0.17–1.23)	0.96 (0.51–1.80)	0.95 (0.64–1.40)	1.05 (0.80–1.36)
**Age**						
18–34	ref.	ref.	ref.	ref.	ref.	ref.
35–49	1.30 (0.41–4.16)	0.68 (0.25–1.80)	0.92 (0.25–3.38)	0.84 (0.39–1.83)	0.49 (0.30–0.78)	0.81 (0.58–1.11)
50+	0.76 (0.22–2.58)	0.31 (0.10–0.92)	1.46 (0.35–6.01)	0.86 (0.32–2.32)	0.46 (0.28–0.77)	0.54 (0.38–0.78)
**Race/ethnicity**						
Non–Hispanic white	ref.	ref.	ref.	ref.	ref.	ref.
Non–white ^b^	0.68 (0.21–2.24)	0.40 (0.10–1.60)	0.83 (0.24–2.87)	1.46 (0.71–2.93)	1.30 (0.84–2.01)	0.73 (0.52–1.03)
**No. of children**						
0	ref.	ref.	ref.	ref.	ref.	ref.
1 or more	0.58 (0.22–1.52)	0.56 (0.24–1.32)	1.51 (0.46–4.99)	0.86 (0.39–1.90)	0.90 (0.58–1.38)	1.58 (1.19–2.09)
**Educational status**						
Not a college graduate	ref.	ref.	ref.	ref.	ref.	ref.
College graduate or more	0.29 (0.10–0.80)	1.68 (0.69–4.09)	0.94 (0.32–2.74)	1.12 (0.53–2.36)	0.46 (0.30–0.71)	1.48 (1.02–2.13)
**Job status**						
Employed or student	ref.	ref.	ref.	ref.	ref.	ref.
Unemployed or other ^c^	0.11 (0.02–0.58)	0.65 (0.23–1.83)	2.07 (0.67–6.38)	0.92 (0.38–2.23)	0.61 (0.34–1.07)	0.84 (0.59–1.19)
**Disability status**						
None	ref.	ref.	ref.	ref.	ref.	ref.
1 or more	1.51 (0.37–6.19)	0.71 (0.19–2.74)	1.27 (0.36–4.54)	1.08 (0.46–2.53)	0.89 (0.42–1.88)	0.95 (0.58–1.56)
**Body mass index**						
Normal	ref.	ref.	ref.	ref.	ref.	ref.
Overweight or obese	0.45 (0.16–1.21)	1.55 (0.64–3.75)	1.23 (0.46–3.33)	1.07 (0.58–1.96)	0.62 (0.43–0.90)	1.05 (0.81–1.35)
**Comorbid condition[s]**						
None	ref.	ref.	ref.	ref.	ref.	ref.
1 or more	1.99 (0.73–5.46)	0.72 (0.31–1.65)	1.79 (0.63–5.10)	0.80 (0.41–1.56)	0.83 (0.56–1.24)	0.94 (0.72–1.22)
**Local stay–at–home order duration**						
Weeks	1.21 (0.74–1.98)	1.05 (0.69–1.62)	1.16 (0.72–1.88)	0.95 (0.70–1.29)	1.24 (1.02–1.51)	0.94 (0.82–1.08)
**Time spent at home**						
Hours	1.05 (0.92–1.18)	1.00 (0.90–1.10)	1.06 (0.89–1.25)	1.08 (0.97–1.20)	1.10 (1.03–1.18)	1.03 (0.99–1.07)
**Depression score ^d^**						
None or mild	ref.	ref.	ref.	ref.	ref.	ref.
Moderate or severe	1.51 (0.49–4.71)	2.58 (1.00–6.63)	2.24 (0.75–6.71)	3.15 (1.58–6.25)	1.58 (0.96–2.62)	2.24 (2.41–4.64)

Note: ^a^ All estimates are mutually adjusted for all other factors under study. ^b^ Includes American Indian/Alaska Native, Asian, Native Hawaiian or other Pacific Islander, Black or African American, more than one race, and unknown/not reported. ^c^ Includes homemaker/stay-at-home parent, retired, and unable to work. ^d^ Estimated using the Patient Health Questionnaire-9 (PHQ-9) and categorized based on 0–5: none/mild; >5: moderate or severe.

**Table 4:** Relative odds of changing positive health behaviors among a sample of US adults during the COVID-19 pandemic and subsequent “Stay-at-Home” orders, 2020.

	Relative ^a^ Odds of Changing Health Behavior, OR (95% CI)			
	Sleep Quality		Physical Activity	
	Worsened	Improved	Decreased	Increased
**Sex**				
Male	ref.	ref.	ref.	ref.
Female	1.42 (1.11–1.83)	1.33 (0.93–1.92)	1.28 (1.00–1.64)	1.47 (1.12–1.93)
**Age**				
18–34	ref.	ref.	ref.	ref.
35–49	1.13 (0.84–1.52)	1.27 (0.83–1.94)	1.01 (0.74–1.37)	1.00 (0.72–1.38)
50+	0.79 (0.57–1.09)	0.62 (0.38–1.02)	0.94 (0.68–1.30)	0.89 (0.63–1.27)
**Race/ethnicity**				
Non-Hispanic white	ref.	ref.	ref.	ref.
Non-white ^b^	1.22 (0.91–1.63)	1.46 (0.98–2.16)	1.29 (0.95–1.73)	1.01 (0.73–1.42)
**No. of children**				
0	ref.	ref.	ref.	ref.
1 or more	1.13 (0.87–1.46)	0.83 (0.57–1.21)	0.78 (0.60–1.03)	1.42 (1.07–1.90)
**Educational status**				
Not a college graduate	ref.	ref.	ref.	ref.
College graduate or more	1.42 (1.04–1.94)	1.04 (0.66–1.63)	0.85 (0.63–1.15)	1.21 (0.84–1.72)
**Job status**				
Employed or student	ref.	ref.	ref.	ref.
Unemployed or other ^c^	0.82 (0.60–1.11)	0.53 (0.31–0.90)	1.06 (0.78–1.43)	0.73 (0.52–1.05)
**Disability status**				
None	ref.	ref.	ref.	ref.
1 or more	1.03 (0.68–1.57)	1.27 (0.68–2.37)	1.32 (0.86–2.03)	0.74 (0.42–1.28)
**Body mass index**				
Normal	ref.	ref.	ref.	ref.
Overweight or obese	1.00 (0.79–1.27)	0.98 (0.70–1.38)	1.11 (0.87–1.41)	0.80 (0.62–1.04)
**Comorbid condition[s]**				
None	ref.	ref.	ref.	ref.
1 or more	1.35 (1.06–1.71)	1.05 (0.74–1.51)	0.98 (0.77–1.26)	0.90 (0.69–1.18)
**Local “Stay-at-Home” order duration**				
Weeks	0.90 (0.79–1.02)	1.03 (0.86–1.23)	1.08 (0.95–1.23)	0.90 (0.78–1.04)
**Time spent at home**				
Hours	1.02 (0.99–1.05)	1.06 (1.00–1.12)	1.06 (1.02–1.09)	1.02 (0.99–1.06)
**Depression score ^d^**				
None or mild	ref.	ref.	ref.	ref.
Moderate or severe	5.32 (4.01–7.06)	0.95 (0.55–1.64)	5.32 (3.73–7.58)	2.42 (1.61–3.64)

Note: ^a^ All estimates are mutually adjusted for all other factors under study. ^b^ Includes American Indian/Alaska Native, Asian, Native Hawaiian or other Pacific Islander, Black or African American, more than one race, and unknown/not reported. ^c^ Includes homemaker/stay-at-home parent, retired, and unable to work. ^d^ Estimated using the Patient Health Questionnaire-9 (PHQ-9) and categorized based on 0–5: none/mild; >5: moderate or severe.

**Table 5:** Behavioral change motivations among a sample of US adults for increasing and decreasing positive and negative health behaviors during the COVID-19 pandemic and subsequent “Stay-at-Home” orders, 2020.

Behavioral Change and Motivations	N (%)	*n* (%)
**Total reasons given for change**	6065 (100.0)	
**Decrease negative health behaviors ^a^**	316 (5.2)	316 (100.0)
More responsibility	31 (0.5)	31 (9.8)
Less time available	30 (0.5)	30 (9.5)
Resource concerns ^b^	58 (1.0)	58 (18.4)
Health concerns ^c^	107 (1.8)	107 (33.9)
Other	90 (1.5)	90 (28.5)
**Increase negative health behaviors ^a^**	1597 (26.3)	1597 (100.0)
Less responsibility	215 (3.5)	215 (13.5)
More time available	431 (7.1)	431 (27.0)
More worried ^d^	242 (4.0)	242 (15.2)
Lonely/unhappy	227 (3.7)	227 (14.2)
Boredom	375 (6.2)	375 (23.5)
Other	107 (1.8)	107 (6.7)
**Increase positive health behaviors ^e^**	2216 (36.5)	2216 (100.0)
More time available	930 (15.3)	930 (42.0)
Boredom	254 (4.2)	254 (11.5)
Social connection	230 (3.8)	230 (10.4)
Stress relief	419 (6.9)	419 (18.9)
Health concerns	334 (5.5)	334 (15.1)
Other	49 (0.8)	49 (2.2)
**Decrease positive health behaviors ^e^**	1936 (31.9)	1936 (100.0)
Less time available	221 (3.6)	221 (11.4)
Less motivation	434 (7.1)	434 (22.4)
More worried/stressed	599 (9.9)	599 (30.9)
Resource concerns ^f^	454 (7.5)	454 (23.5)
Illness	7 (0.1)	7 (0.4)
Other	221 (3.6)	221 (11.4)

Note: ^a^ Includes tobacco use, drug use, and alcohol consumption. ^b^ Includes financial concerns and concerns about limited locations available to engage in negative health behaviors. ^c^ Includes maintaining/improving health, and due to illness. ^d^ Includes worries about health, finances, and job security. ^e^ Includes physical activity and sleep quality. ^f^ Includes concerns about locations to engage in physical activity.

## Supplementary Materials

The following are available online at https://www.mdpi.com/1660-4601/17/17/6268/s1. Table S1: Characteristics of a sample of US adults changing negative health behaviors during the COVID-19 pandemic. Table S2: Characteristics of a sample of US adults changing positive health behaviors during the COVID-19 pandemic. Table S3: Crude odds of a sample of US adults changing negative health behaviors during the COVID-19 pandemic. Table S4: Crude odds of a sample of US adults changing positive health behaviors during the COVID-19 pandemic.
