# Supplementary material for: Health Behavior Changes During COVID-19 Pandemic and Subsequent “Stay-at-Home” Orders
Source: Int J Environ Res Public Health. 2020 Aug 28;17(17):6268. doi: 10.3390/ijerph17176268 (PMC7504386; doi:10.3390/ijerph17176268)
Supplement: Supplementary file 1 [file ijerph-17-06268-s001.pdf]

Table S1. Descriptive statistics of positive and negative health behaviors US adults engaged in during COVID-19 pandemic and subsequent "Stay-at-Home" orders and policies, 2020.

Health behavior	Estimate
**Tobacco use, n (%)**	
Never	1,352 (74.7)
Former	279 (15.4)
Current[a]	178 (9.8)
**Marijuana use, n (%)**	
Never	700 (38.7)
Former	879 (48.6)
Current[a]	230 (12.7)
**Alcohol consumption per day, n (%)**	
Zero drinks	598 (33.1)
1 drink	393 (21.7)
2 drinks	382 (21.1)
3 drinks	193 (10.7)
4 drinks or more	243 (13.4)
**Physical activity**	
MET minutes per week, median (IQR)	2,034 (891.0-4,464.0)
Low levels, n (%)	433 (23.9)
Moderate levels, n (%)	554 (30.6)
High levels, n (%)	822 (45.4)
**Sleep index & quality**	
PSI, mean (SD)	6.5 (3.3)
Very good, n (%)	255 (14.1)
Fairly good, n (%)	1,050 (58.0)
Fairly bad, n (%)	421 (23.3)
Very bad, n (%)	83 (4.6)

Abbreviations: MET, metabolic equivalent of task; IQR, interquartile range; PSI, Pittsburgh Sleep Index; SD, standard deviation. Note: [a] Includes using the substance on at least 1-day within the past 30-days.

Formatted: Superscript

Formatted: Superscript

Table ~~S1~~S2. Characteristics of a sample of US adults changing negative health behaviors during the COVID-19 pandemic

	**Tobacco use, n (%)**				**Marijuana use, n (%)**				**Alcohol consumption**			
	**Increased**	**Decreased**	**Same**	***P***	**Increased**	**Decreased**	**Same**	***P***	**Increased**	**Decreased**	**Same**	***P***
**Overall**	54 (30.5)	34 (19.2)	89 (50.3)	<0.01	84 (36.5)	24 (10.4)	122 (53.0)	<0.01	521 (38.5)	161 (11.9)	672 (49.6)	<0.01
**Sex**												
Male	22 (22.2)	23 (23.2)	54 (54.6)	0.02	32 (33.3)	13 (13.5)	51 (53.1)	0.37	169 (35.9)	55 (11.7)	247 (52.4)	0.30
Female	32 (41.0)	11 (14.1)	35 (44.9)		52 (38.8)	11 (8.2)	71 (53.0)		352 (29.9)	106 (12.0)	425 (48.1)	
**Age**												
18-34	19 (36.5)	13 (25.0)	20 (38.50	0.15	46 (42.2)	8 (7.3)	55 (50.5)	0.20	165 (39.3)	80 (19.1)	175 (41.7)	<0.01
35-49	25 (32.9)	13 (17.1)	38 (50.0)		28 (33.3)	9 (10.7)	47 (56.0)		256 (44.7)	46 (8.0)	271 (47.3)	
50+	10 (20.4)	8 (16.3)	31 (63.3)		10 (27.0)	7 (18.9)	20 (54.1)		100 (27.7)	35 (9.7)	226 (62.6)	
**Race/ethnicity**												
NH-white	51 (33.3)	28 (18.3)	74 (48.4)	0.10	63 (34.8)	20 (11.1)	98 (54.1)	0.62	450 (39.9)	118 (10.5)	561 (49.7)	<0.01
Non-white[a]	3 (12.5)	6 (25.0)	15 (62.5)		21 (42.9)	4 (8.2)	24 (49.0)		71 (31.6)	43 (19.1)	111 (49.3)	
**No. of children**												
0	29 (32.2)	18 (20.0)	43 (47.8)	0.79	58 (38.2)	14 (9.2)	80 (52.6)	0.61	244 (32.5)	107 (14.3)	399 (53.2)	<0.01
1 or more	25 (28.7)	16 (18.4)	46 (52.9)		26 (33.3)	10 (12.8)	42 (53.9)		277 (45.9)	54 (8.9)	273 (45.2)	
**Educational status**												
Not a college grad	14 (23.3)	16 (26.7)	30 (50.0)	0.13	19 (33.3)	8 (14.0)	30 (52.6)	0.56	58 (28.3)	44 (21.5)	103 (50.2)	<0.01
College grad/more	40 (34.2)	18 (15.4)	59 (50.4)		65 (37.6)	16 (9.3)	92 (53.2)		463 (40.3)	117 (10.2)	569 (49.5)	
**Job status**												
Employed/student	46 (31.9)	32 (22.2)	66 (45.8)	0.03	72 (37.7)	16 (8.4)	103 (53.9)	0.08	446 (39.4)	143 (12.6)	544 (48.0)	0.02
Unemployed/other	8 (24.2)	2 (6.1)	23 (69.7)		12 (30.8)	8 (20.5)	19 (48.7)		75 (33.9)	18 (8.1)	128 (57.9)	
**Disability status**												
None	49 (30.8)	29 (18.2)	81 (50.9)	0.57	70 (35.9)	19 (9.7)	106 (54.4)	0.51	484 (38.3)	151 (12.0)	629 (49.8)	0.87
1 or more	5 (27.8)	5 (27.8)	8 (44.4)		14 (40.0)	5 (14.3)	16 (45.7)		37 (41.1)	10 (11.1)	43 (47.8)	
**Body mass index**												
Normal	15 (27.8)	14 (25.9)	25 (46.3)	0.32	39 (37.5)	8 (7.7)	57 (54.8)	0.47	200 (35.7)	91 (16.3)	269 (48.0)	<0.01
Overwt/obese	39 (31.7)	20 (16.3)	64 (52.0)		45 (35.7)	16 (12.7)	65 (51.6)		321 (40.4)	70 (8.8)	403 (50.8)	
**Comorbid condition[s]**												
None	33 (32.0)	19 (18.5)	51 (49.5)	0.86	52 (38.2)	10 (7.4)	74 (54.4)	0.18	319 (37.9)	110 (13.1)	412 (49.0)	0.22
1 or more	21 (28.4)	15 (20.3)	38 (51.4)		32 (34.0)	14 (14.9)	48 (51.1)		202 (39.4)	51 (9.9)	260 (50.7)	
**Local stay-at-home order duration**												
Wks, mean (SD)	3.8 (1.1)	3.9 (1.0)	3.7 (0.8)	0.61	4.1 (1.0)	4.2 (0.9)	4.1 (0.9)	0.79	3.8 (0.9)	4.1 (0.9)	3.8 (0.9)	<0.01
**Time spent at home**												
Hrs, median (IQR)	23.0	23.0	21.0	0.24	22.5	22.0	22.0	0.36	23.0	23.0	22.0	<0.01

	(20.0-23.0)	(20.0-23.0)	(20.0-23.0)		(21.0-23.0)	(21.0-23.5)	(20.0-23.0)		(22.0-23.0)	(22.0-24.0)	(21.0-23.0)	
**Depression score**												
None or mild	37 (26.8)	25 (18.1)	76 (55.1)	0.05	50 (30.1)	16 (9.6)	100 (60.2)	<0.01	368 (33.5)	131 (11.9)	599 (54.6)	<0.01
Moderate or severe	17 (43.6)	9 (23.1)	13 (33.3)		34 (53.1)	8 (12.5)	22 (34.4)		153 (59.8)	30 (11.7)	73 (28.5)	

Abbreviations: grad, graduate; Hrs, hours; IQR, interquartile range; NH-, non-Hispanic; Overwt, overweight; SD, standard deviation; Wks, weeks.

Note: [a] Includes American Indian/Alaska Native, Asian, Native Hawaiian or other Pacific Islander, Black or African American, more than one race, and unknown/not reported. [b] Includes homemaker/stay-at-home parent, retired, and unable to work [c] Estimated using the Patient Health Questionnaire-9 (PHQ-9), and categorized based on 0-5: none/mild; >5: moderate or severe.

Table ~~S2~~S3. Characteristics of a sample of US adults changing positive health behaviors during the COVID-19 pandemic.

	Sleep quality, n (%)				Physical activity, n (%)			
	**Improved**	**Worsened**	**Same**	***P***	**Increased**	**Decreased**	**Same**	***P***
**Overall**	175 (9.7)	560 (31.0)	1074 (59.4)	<0.01	455 (25.2)	706 (39.0)	648 (35.8)	<0.01
**Sex**								
Male	53 (9.0)	140 (23.8)	396 (67.2)	<0.01	131 (22.2)	205 (34.8)	253 (43.0)	<0.01
Female	122 (10.0)	420 (34.4)	678 (55.6)		324 (26.6)	501 (41.1)	395 (32.4)	
**Age**								
18-34	62 (10.9)	188 (33.0)	320 (56.1)	<0.01	142 (24.9)	240 (42.1)	188 (33.0)	0.01
35-49	79 (11.0)	247 (34.3)	394 (54.7)		206 (28.6)	261 (36.3)	253 (35.1)	
50+	34 (6.6)	125 (24.1)	360 (69.4)		107 (20.6)	205 (39.5)	207 (39.9)	
**Race/ethnicity**								
NH-white	132 (8.9)	452 (30.5)	899 (60.6)	0.02	378 (25.5)	562 (37.9)	543 (36.6)	0.11
Non-white[a]	43 (13.2)	108 (33.1)	175 (53.7)		77 (23.6)	144 (44.2)	105 (32.2)	
**No. of children**								
0	100 (9.8)	296 (29.0)	626 (61.3)	0.11	215 (21.0)	437 (42.8)	370 (36.2)	<0.01
1 or more	75 (9.5)	264 (33.6)	448 (56.9)		240 (30.5)	269 (34.2)	278 (35.3)	
**Educational status**								
Not a college grad	27 (8.9)	85 (28.2)	190 (62.9)	0.39	59 (19.5)	140 (46.4)	103 (34.1)	<0.01
College grad/more	148 (9.8)	475 (31.5)	884 (58.7)		396 (26.3)	566 (37.6)	545 (36.2)	
**Job status**								
Employed/student	156 (10.6)	462 (31.4)	852 (58.0)	<0.01	389 (26.5)	556 (37.8)	525 (35.7)	0.02
Unemployed/other	19 (5.6)	98 (28.9)	222 (65.5)		66 (19.5)	150 (44.3)	123 (36.3)	
**Disability status**								
None	161 (9.7)	504 (30.2)	1004 (60.2)	0.04	433 (25.9)	627 (37.6)	609 (36.5)	<0.01
1 or more	14 (10.0)	56 (40.0)	70 (50.0)		22 (15.7)	79 (56.4)	39 (27.9)	
**Body mass index**								
Normal	77 (10.4)	223 (30.2)	438 (59.4)	0.62	210 (28.5)	271 (36.7)	257 (34.8)	0.02
Overwt/obese	98 (9.2)	337 (31.5)	636 (59.4)		245 (22.9)	435 (40.6)	391 (36.5)	
**Comorbid condition[s]**								
None	115 (10.5)	295 (26.8)	690 (62.7)	<0.01	297 (27.0)	396 (36.0)	407 (37.0)	<0.01
1 or more	60 (8.5)	265 (37.4)	384 (54.2)		158 (22.3)	310 (43.7)	241 (34.0)	
**Local stay-at-home order duration**								
Wks, mean (SD)	3.9 (0.9)	3.8 (1.0)	3.9 (0.8)	0.26	3.8 (0.9)	3.9 (0.9)	3.8 (0.8)	0.02
**Time spent at home**								
Hrs, median (IQR)	23.0 (22.0-23.0)	23.0 (22.0-23.0)	22.0 (21.0-23.0)	<0.01	23.0 (22.0-23.0)	23.0 (22.0-24.0)	22.0 (20.0-23.0)	<0.01
**Depression score**								
None or mild	157 (10.7)	345 (23.4)	972 (65.9)	<0.01	383 (26.0)	489 (33.2)	602 (40.8)	<0.01
Moderate or severe	18 (5.4)	215 (64.2)	102 (30.5)		72 (21.5)	217 (64.8)	46 (13.7)	

Abbreviations: grad, graduate; Hrs, hours; IQR, interquartile range; NH-, non-Hispanic; Overwt, overweight; SD, standard deviation; Wks, weeks.

Note: [a] Includes American Indian/Alaska Native, Asian, Native Hawaiian or other Pacific Islander, Black or African American, more than one race, and unknown/not reported. [b] Includes homemaker/stay-at-home parent, retired, and unable to work [c] Estimated using the Patient Health Questionnaire-9 (PHQ-9), and categorized based on 0-5: none/mild; >5: moderate or severe

Table ~~S3~~S4. Reasons for changing negative health behaviors.

Negative health behavior change and motivations	N (%)	n (%)
**Total reasons given for change**	1,913 (100.0)	
**Tobacco use - decreased**	63 (3.3)	63 (100.0)
More responsibility	8 (0.4)	8 (12.7)
Less time available	4 (0.2)	4 (6.3)
Less places to use tobacco	6 (0.3)	6 (9.5)
Don't want to spend money on tobacco	13 (0.7)	13 (20.6)
Maintain health	24 (1.3)	24 (38.1)
Illness	0 (0.0)	0 (0.0)
Other	8 (0.4)	8 (12.7)
**Marijuana use - decreased**	29 (1.5)	29 (100.0)
More responsibility	3 (0.2)	3 (10.3)
Less time available	1 (0.1)	1 (3.4)
Less places to use marijuana	3 (0.2)	3 (10.3)
Don't want to spend money on marijuana	3 (0.2)	3 (10.3)
Maintain health	8 (0.4)	8 (27.6)
Illness	0 (0.0)	0 (0.0)
Other	11 (0.6)	11 (37.9)
**Alcohol consumption - decreased**	224 (11.7)	224 (100.0)
More responsibility	20 (1.0)	20 (8.9)
Less time available	25 (1.3)	25 (11.2)
Don't want to spend money on alcohol	33 (1.7)	33 (14.7)
Maintain health	75 (3.9)	75 (33.5)
Other	71 (3.7)	71 (31.7)
**Tobacco use - increased**	119 (6.2)	119 (100.0)
Less responsibility	4 (0.2)	4 (3.4)
More time available	33 (1.7)	33 (27.7)
Unhappiness	9 (0.5)	9 (7.6)
Worry/concern about health	16 (0.8)	16 (13.4)
Worry/concern about job security	9 (0.5)	9 (7.6)
Loneliness	9 (0.5)	9 (7.6)
Boredom	31 (1.6)	31 (26.1)
Other	8 (0.4)	8 (6.7)
**Marijuana use - increased**	246 (12.9)	246 (100.0)
Less responsibility	35 (1.8)	35 (14.2)
More time available	51 (2.7)	51 (20.7)
Unhappiness	24 (1.3)	24 (9.8)
Worry/concern about health	21 (1.1)	21 (8.5)
Worry/concern about job security	13 (0.7)	13 (5.3)
Loneliness	25 (1.3)	25 (10.2)
Boredom	57 (3.0)	57 (23.2)

Other	20 (1.0)	20 (8.1)
**Alcohol consumption - increased**	1232 (64.4)	1232 (100.0)
Less responsibility	176 (9.2)	176 (14.3)
More time available	347 (18.1)	347 (28.2)
Unhappiness	89 (4.7)	89 (7.2)
Worry/concern about health	113 (5.9)	113 (9.2)
Worry/concern about job security	70 (3.7)	70 (5.7)
Loneliness	71 (3.7)	71 (5.8)
Boredom	287 (15.0)	287 (23.3)
Other	79 (4.1)	79 (6.4)

Table ~~S4~~S5. Reasons for changing positive health behaviors during COVID-19 pandemic.

Positive behavior change and motivations	N (%)	n (%)
**Total reasons given for change**	4,152 (100.0)	
**Physical activity - increased**	1,499 (36.1)	1,499 (100.0)
More time available	359 (8.6)	359 (23.9)
Boredom	254 (6.1)	254 (16.9)
Social connection	230 (5.5)	230 (15.3)
Stress relief	305 (7.3)	305 (20.3)
Health concerns	334 (8.0)	334 (22.3)
Other	17 (0.4)	17 (1.1)
**Sleep quality - improved**	717 (17.3)	717 (100.0)
More time available	421 (10.1)	421 (58.7)
Less work responsibility/time	150 (3.6)	150 (20.9)
Stress	114 (2.7)	114 (15.9)
Other	32 (0.8)	32 (4.5)
**Physical activity - decreased**	1361 (32.8)	1,361 (100.0)
Less time available	112 (2.7)	112 (8.2)
Less motivation	434 (10.5)	434 (31.9)
More worried/stressed	229 (5.5)	229 (16.8)
Lack of locations to be active	327 (7.9)	327 (24.0)
Lack of persons to be active with	127 (3.1)	127 (9.3)
Illness	6 (0.1)	6 (0.4)
Other	126 (3.0)	126 (9.3)
**Sleep quality - worsened**	575 (13.8)	575 (100.0)
Less time available	33 (0.8)	33 (5.7)
More work responsibility/time	76 (1.8)	76 (13.2)
More worried/stressed	370 (8.9)	370 (64.4)
Illness	1 (0.0)	1 (0.2)
Other	95 (2.3)	95 (16.5)
